# Serum Lipid Profile in Polish Women Adhering to Different Dietary Patterns: The Cardioprotective Potential of Plant-Based Diets

**DOI:** 10.3390/nu17213381

**Published:** 2025-10-28

**Authors:** Patrycja Gogga, Patrycja Szulc, Agata Janczy

**Affiliations:** Division of Food Commodity Science, Department of Clinical Nutrition, Faculty of Health Sciences, Medical University of Gdańsk, ul. Dębinki 7, 80-211 Gdańsk, Poland; patkaszulc@gumed.edu.pl (P.S.); agata.janczy@gumed.edu.pl (A.J.)

**Keywords:** lipid profile, cholesterol, cardiovascular diseases, vegetarian diet, vegan diet

## Abstract

**Background/Objectives:** Cardiovascular diseases (CVDs) remain the leading cause of mortality worldwide, with elevated low-density lipoprotein cholesterol (LDL-C) and total cholesterol (TC) being major risk factors. Diet is a key modulator of these parameters, and healthful plant-based diets—popular particularly among women—are associated with cardiovascular benefits. The present study aimed to evaluate the serum lipid profile and to identify dietary components associated with differences in lipid fractions in healthy women adhering to different dietary patterns. **Methods:** This was a cross-sectional, single-center, convenience sample study of 128 healthy women of similar age, normal BMI, and comparable body fat, allocated to four dietary groups: vegans (*n* = 45), lacto-ovo-vegetarians (*n* = 52), pescatarians (*n* = 12), and omnivores (*n* = 19). Serum lipid profiles were determined using enzymatic kits. Intake of selected nutrients was assessed based on 7-day dietary records. Physical activity was estimated using the physical activity level (PAL) index. Analyses included different ANOVA approaches and PCA. **Results:** Omnivores exhibited the highest serum concentrations of LDL-C, TC, and TGs compared with other dietary groups. A significant association was noted between elevated TC and higher intake of saturated fatty acids (SFAs), cholesterol, and animal protein, accompanied by lower intake of fiber and plant protein. Additionally, women with lower TGs and higher HDL-C showed lower PAL values. No significant differences in HDL-C concentrations were observed between groups. **Conclusions:** Plant-based diets, defined by lower consumption of SFAs, cholesterol, and animal protein alongside higher intake of fiber and plant protein, were associated with a more favorable lipid profile. These findings support the role of vegetarian and vegan diets in CVDs prevention and management, particularly when coupled with regular physical activity; however, further interventional studies among diverse populations are necessary to confirm our results.

## 1. Introduction

Cardiovascular diseases (CVDs) represent one of the most serious life-threatening conditions in the world. According to data from the World Health Organization (WHO), in 2022 they led to the deaths of 19.8 million people [1]. Moreover, according to the World Heart Federation (WHF), CVDs account for about 35% of all deaths among women and remain their leading cause of death [2]. The most common CVDs are coronary artery disease, a condition affecting blood vessels that supply the heart muscle, and cerebrovascular disease, a condition affecting blood vessels that supply the brain [1].

Atherosclerosis, a progressive, inflammatory disease of the arteries marked by the accumulation of lipids and fibrous elements within the arterial walls, is the foremost cause of CVDs, as it reduces blood flow [3,4]. Although atherosclerosis can develop in any part of the arterial system, it poses the greatest risk when plaques occur in the arteries supplying the heart or brain. However, when it affects the peripheral arteries, it may result in peripheral artery disease, which further elevates the risk of myocardial infarction and cerebrovascular events [5,6].

While the precise cause of atherosclerosis remains unclear, several factors are known to contribute to its development. These include sedentary lifestyle, poor diet, ageing, psychological and socioeconomic influences, and—above all—an impaired lipid profile, which is a laboratory test assessing the bloodstream concentrations of certain lipids, commonly used for estimating cardiovascular risk [7]. The standard lipid profile includes total cholesterol (TC), triglycerides (TGs), high-density lipoprotein cholesterol (HDL-C), low-density lipoprotein cholesterol (LDL-C), and non-HDL cholesterol (non-HDL-C) [8]; however, non-HDL-C and LDL-C have been considered equivalent markers [9]. The 2024 guidelines for the laboratory diagnosis of lipid metabolism disorders, developed by the Polish Society of Laboratory Diagnostics (PSLD) and the Polish Lipid Association (PoLA), propose the following reference ranges for the standard lipid profile in healthy subjects:

- Fasting and non-fasting TC < 190 mg/dL;
- Fasting and non-fasting LDL-C < 115 mg/dL;
- Fasting and non-fasting HDL-C: female > 45 mg/dL, male > 40 mg/dL;
- Fasting TGs < 100 mg/dL [9].

Abnormal lipid levels in the blood, known as dyslipidemia, are considered one of the main risk factors for CVDs development, as they contribute to the atherosclerotic process [10]. Particularly critical in the formation of atheromatous plaques are elevated levels of LDL-C, making this lipoprotein a key element in the diagnosis, monitoring, and treatment of lipid disorders, as well as in the prediction of cardiovascular events [8] or ischemic stroke [11].

In 2019, the American College of Cardiology/American Heart Association (ACC/AHA) recommended a diet based on vegetables, fruits, and whole grains as the best food choice for lowering atherosclerotic CVDs risk, with legumes (along with fish and poultry) as preferred source of protein [12]. Two years later, the European Society of Cardiology (ESC) in its guidelines on the prevention of CVDs, emphasized an important role of using more plant- and less animal-based foods in a healthy, cardioprotective eating pattern [13].

Plant-based diets are increasingly recognized dietary strategies. A vegetarian diet, most commonly referring to a lacto-ovo-vegetarian pattern, excludes meat, fish, and seafood, while a vegan diet eliminates all animal-derived food products. According to the Academy of Nutrition and Dietetics, well-planned vegetarian and vegan diets are nutritionally adequate for adults and may offer health benefits in the prevention and management of various diseases, particularly in reducing cardiometabolic risk (during childhood, pregnancy, and lactation professional guidance is recommended, addressing the unique nutritional requirements of these groups) [14]. Evidence suggests that such eating patterns may improve the lipid profile, particularly by lowering LDL-C and TC levels [15,16,17,18]. Some research also reports increased HDL-C [15] and decreased TGs concentrations [19]. Nevertheless, studies indicate that plant-based diets, through their beneficial effect on the lipid profile, can significantly reduce the risk of CVDs [20].

Previous studies have compared plant-based and omnivorous diets with respect to the lipid profile [15,21,22]. However, to our knowledge, few have combined these comparisons with an analysis of the specific nutritional components that may account for favorable or unfavorable outcomes, particularly within a group of young, healthy women. The aim of the study was to examine the serum lipid profile in a group of women homogeneous in terms of age, BMI, and body fat, and to identify which dietary factors contribute the most to the observed differences in lipid fractions between omnivores and plant-based diet users.

## 2. Materials and Methods

### 2.1. Participants

The present research is a cross-sectional, single-center study based on a convenience sample. It is a part of the project: “The influence of meat-free diet on serum levels of leptin and obesity markers, and gut microbiome in vegetarian and vegan subjects” (no: 01-0293/08/316) and has received approval from the local Independent Bioethics Commission for Research (no: NKBBN/234/2016). From all project participants, 128 apparently healthy women with normal BMI (18.50–24.99 kg/m^2^) were chosen—45 vegans (VEGs), 52 lacto-ovo-vegetarians (LOVs), 12 pescatarians (PSCs), and 19 omnivores (OMNs)—for the lipid profile analysis. The diet of VEG volunteers consisted only of plant-based products. The LOV group encompassed women who—apart from plant-derived foods—consumed eggs (5), dairy (6), or both food groups (41). PSCs used eggs, dairy, and fish in their diets, while OMNs consumed also meat, and did not exclude any animal-derived products.

Participants were recruited via social media. Information about the study, its purpose, and research procedure, was released on Facebook, with instructions on how to join in. The inclusion criteria for the study groups were as follows: adherence to a particular diet for at least three consecutive months, age ≥ 18 years, absence of chronic diseases and eating disorders, and not being pregnant or lactating. Participants in the OMN group were required to consume meat regularly (at least several times per week) and to meet the same additional criteria as the study groups. All women were non-smokers and did not consume alcohol on a regular basis. All participants agreed voluntarily to take part in the project and provided an informed consent prior to the onset of the study. Subjects underwent two consultations with a well-trained, experienced interviewer. During the first one, they provided detailed information about the specificity and longevity of their diets, and also about their physical activity level and overall health status. Additionally, anthropometric parameters were taken from all participants:

- Body mass (Jawon Medical X-contact 350, Gyeongsan-si, Republic of Korea) and height (stadiometer Seca 213, Hamburg, Germany), both used for calculating the body mass index (BMI);
- The circumference of waist and hips (tape measure Seca 201, Hamburg, Germany), used for calculating the waist-to-hip ratio (WHR);
- Body composition, using the BIA method (Jawon Medical X-contact 350, Gyeongsan-si, Republic of Korea).

Age and basic anthropometric characteristics, as well as time spent following a particular non-meat diet, are shown in Table 1.

In order to estimate the intake of selected nutrients, participants completed dietary records for seven days (five working days and two weekend days). During the first consultation, respondents were instructed on how to record their food intake. They were supposed to precisely record every consumed food and drink (including minor ingredients such as salt, herbs, cooking oils, etc.), using both the International System of Units (grams, liters, etc.) and kitchen measurements (cups, spoons, etc.). For purchased foods, participants were asked to provide the name of the producer and also the precise amount consumed. During the second consultation, home-prepared dietary records were verified by the interviewer, who—if necessary—questioned the respondents about any missing details. Obtained data were analyzed using the dietary intake assessment software Aliant 2.0 (Anmarsoft, Gdańsk, Poland); however, three women did not complete the records of food intake, all using the LOV diet.

Based on the information provided by the participants during the first consultation, physical activity level (PAL) values were assessed. Subsequently, all women were assigned to one of three categories—low physical activity (PAL ≤ 1.69), medium physical activity (PAL 1.7–1.99), or high physical activity (PAL ≥ 2.0) [23]. For principal components analysis (PCA), two of the categories were merged, resulting in groups termed inactive (low activity) and active (moderate or high activity).

### 2.2. Sample Collection and Lipid Profile Analysis

During the second consultation, fasting blood samples were collected from subjects and immediately centrifuged to separate serum, which was stored at −80 °C prior to the analysis. Serum levels of all lipid profile components were determined directly using commercial kits (Alpha Diagnostics, Warsaw, Poland), based on enzymatic colorimetric assays. For HDL-C and LDL-C, non-target lipoproteins were first removed from the samples. Triacylglycerols and cholesterol esters were enzymatically hydrolyzed to release free glycerol and cholesterol, which were subsequently quantified through reactions with kit reagents that yielded colored end products. Their intensity was measured photometrically using a microplate reader (Bio-Rad, California, CA, USA) at 500 nm (TC, TGs) or 600 nm (LDL-C, HDL-C). Final concentrations were calculated using both the ratio of sample to calibrator absorbance and the defined calibrator concentration provided by the manufacturer. According to the manufacturer’s specifications, intra-assay CVs ranged from 0.6% to 2.5%, and inter-assay CVs from 1.3% to 3.0% for all lipid profile parameters.

For the purpose of PCA regarding the possible impact of nutrient intake on circulating lipid levels, participants were divided into two groups for each lipid profile component: within the reference range and above (TGs, TC, LDL-C), or below the reference range (HDL-C) [9].

### 2.3. Statistical Analysis

All basic characteristics of the study participants and comparisons between diet groups were calculated in Statistica 13.3 (TIBCO Software Inc., San Ramon, CA, USA). Values were defined as outliers if they were more than 1.5 times the interquartile range above the third quartile or below the first quartile. Such values were excluded from further analyses. For assessment of the data, distribution histogram analysis and the Shapiro–Wilk test were used. For normally distributed data, one-way analysis of variance (ANOVA) was performed, followed by Tukey’s post hoc test. Homogeneity of variances was assessed using Levene’s test. For groups with unequal variances, Welch’s ANOVA was conducted, with Games–Howell post hoc comparisons. Data with non-normal distributions were analyzed using the Kruskal–Wallis test, followed by Dunn’s post hoc test. In order to compare PAL values between groups, Pearson’s chi-squared test was applied.

PCA and multiple linear regression analyses were performed in an open-source software, Python 3.10.11 (Python Software Foundation License, USA). Libraries used were as follows: pandas, seaborn, NumPy, SciPy, matplotlib, statsmodels, pingouin, and scikit-learn. To analyze the association between dietary patterns and lipid profile, principal component values were compared using one-way ANOVA or Welch’s ANOVA, depending on variance homogeneity, followed by Tukey’s or Games–Howell post hoc tests, respectively. Associations between principal components derived from selected nutrients and lipid profile parameters were assessed using Student’s *t*-test or Welch’s *t*-test, as appropriate.

To examine associations between lipid profile parameters and selected predictors—diet type, BMI (kg/m^2^), age (y), total energy intake (kcal), and physical activity level—multivariable linear regression analyses were performed using the ordinary least squares (OLS) method. Diet type and physical activity level were included as categorical predictors with two levels (omnivorous vs. non-omnivorous; high vs. low). Regression coefficients (β) were estimated by minimizing the sum of squared residuals between observed and predicted values. The overall significance of each model was assessed using the F-test, and the significance of individual predictors using the Student’s *t*-test. Model fit was evaluated with the coefficient of determination (R^2^) and the adjusted R^2^, which accounts for the number of predictors. Each lipid profile parameter was analyzed as a separate dependent variable according to the model: lipid parameter = β_0_ + β_1_(diet type) + β_2_(BMI) + β_3_(age) + β_4_(total energy intake) + β_5_(physical activity level) + ε.

The results of all analyses were considered as statistically significant when *p* < 0.05.

## 3. Results

There were statistically significant differences in LDL-C, TC, and TGs serum levels among diet groups, with the highest values found in the OMN group. No differences in HDL-C concentrations were found (Table 2).

**Figure 1:**
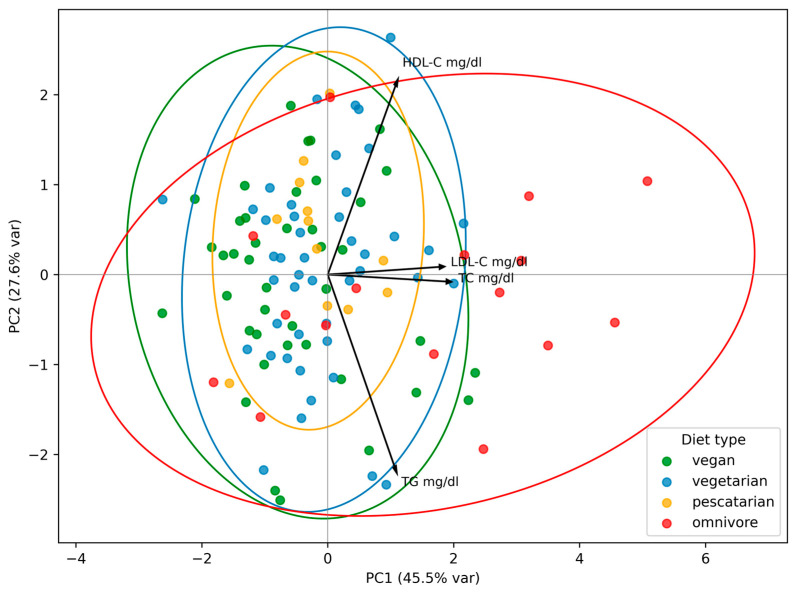
Principal component analysis (PCA) of lipid profile by type of diet. PC1 and PC2 are the first and the second principal component, respectively. The percentage of variance explained by each component is indicated in parentheses. Each point represents an individual participant. Ellipses indicate the grouping of individuals within each dietary pattern.

To identify patterns in lipid profile components across dietary groups, PCA was performed. OMNs were characterized by PC1 values—which explained most of the variance—higher than those of every other dietary group (F = 4.35, *p* = 0.01). PC1 was mainly driven by strong loadings of TC and LDL-C, with additional moderate contributions from TGs and HDL-C. This suggests that non-vegetarians tended to have higher levels of all lipid profile parameters, particularly TC and LDL-C, and were a group separated from all other types of diet. PC2 was characterized by strong opposite loadings for HDL and TGs, indicating an axis of high HDL-C/low TGs versus low HDL-C/high TGs, but this pattern did not differentiate plant-based diet users from OMNs, as PC2 did not reach statistical significance (F = 0.89, *p* = 0.454) (Figure 1).

Statistically significant differences were found in the intake of all analyzed macronutrients, especially for the animal protein, cholesterol, and some fatty acid groups. Notably, energy intake was highly consistent across all dietary groups (Table 3).

To explore the relationships between lipid profile components and dietary factors, PCA was used. For TC, PC1 explained 32.1% of the total variance (t = −2.27, *p* = 0.025) and was primarily defined by moderate positive loadings of dietary SFAs, cholesterol, and animal protein, suggesting that higher PC1 scores reflected diets rich primarily in these nutrients. MUFAs and energy derived from protein contributed with weak positive loadings, whereas fiber and plant protein exhibited weak negative loadings. PC2 accounted for an additional 27.4% of the variance and was also statistically significant (t = −3.09, *p* = 0.003), driven mainly by moderate positive loadings for energy derived from protein and fat. For LDL-C, PC1 and PC2 accounted for 31.8% and 27.5% of the variance, respectively. Both components represented dietary patterns analogous to those identified for TC. PC2 was statistically significant (t = −2.80, *p* = 0.006), whereas PC1 showed a non-significant trend (t = −1.54, *p* = 0.125). Additionally, PC3, which explained 17.5% of variance, was also significant (t = 2.07, *p* = 0.041), mainly driven by a moderate negative loading for energy derived from carbohydrates. The results suggest that diet components—mainly cholesterol, SFAs, and animal protein—have influence on lipid profile, especially on TC values and likely on LDL-C values.

In accordance with the result of ANOVA showing no differences in HDL-C concentrations across dietary groups, PCA did not reveal meaningful associations between this parameter and the intake of selected nutrients. Although PC1 explained 32.3% of the variance (t = −0.237, *p* = 0.828) and PC2 captured an additional 27.5% (t = −1.518, *p* = 0.132), both components failed to reach statistical significance.

For TGs, PC1 explained 31.8% of the variance and represented a dietary pattern high in SFAs, cholesterol, and animal protein, and low in fiber and plant protein; however, this result showed only a non-significant trend (t = 1.526, *p* = 0.131). PC2 explained 27.4% of the variance but was not significant (t = 0.839, *p* = 0.404).

There were no differences in PAL values among users of different dietary patterns (χ2 = 2.01, *p* = 0.570). However, when considering merged groups (low vs. moderate/high activity), subjects with higher activity showed higher PC2 values (t = 2.13, *p* = 0.035), which accounted for 27.6% of variance and were characterized by a strong positive loading with HDL-C and a strong negative loading with TGs. Subjects with higher PC1 values, which accounted for 45.5% of variance, tended to be less physically active and to have higher TC and LDL-C levels; however, this association did not reach statistical significance (t = −1.15, *p* = 0.271).

The regression models indicated that diet type was the most consistent and significant predictor across lipid parameters, with an omnivorous diet associated with higher TC, LDL-C, and TGs levels. Age and BMI were positively associated with TC levels, whereas higher level of physical activity showed a protective effect, reflected by lower concentrations of this parameter. No significant associations were observed between HDL-C and any of the tested predictors (Table 4).

## 4. Discussion

The study aimed to assess the lipid profile in women following meat-including and various types of plant-based diets, and to identify the key food components affecting circulating lipids. As expected, LDL-C concentrations were highest in the OMN group, followed by the PSC group, with LOV and VEG groups exhibiting the lowest values; this difference was statistically significant. TC was also significantly higher in OMNs compared with VEGs, in line with previous findings [15,17,21,22,24,25].

No statistically significant differences were observed in HDL-C levels, similar to some meta-analyses [26,27]. However, several studies report that vegetarian and vegan diets slightly decrease HDL-C [28,29,30], while the others observe higher concentrations in vegetarians compared to omnivores [15,17]. This inconsistency probably reflects its stronger dependence on non-dietary factors, such as physical activity, sex, or genetics [31,32,33]. Accordingly, PCA did not reveal any associations with dietary components, and the regression analysis showed no effect of diet type on HDL-C concentrations. Moreover, it is important to note that HDL-C shows a nonlinear relationship with CVD risk; beyond a certain threshold, further increase is not associated with additional risk reduction and may even be harmful [34,35].

OMNs presented significantly higher TGs levels compared to PSCs and VEGs. Generally, the literature on serum TGs concentrations presents mixed results. Similarly to HDL-C, some studies report higher TGs in omnivores compared to vegetarians or vegans [15,22], whereas others find no such association [17,36,37], or observe even higher TGs levels in plant-based diet users [29,38]. It is well established that the regulation of TGs levels is influenced not only by dietary factors but also by a range of other determinants, both modifiable (body weight, physical activity, alcohol consumption, smoking) and non-modifiable, including genetic predisposition [9]. Oh et al. [39] demonstrated that maintaining a healthy body weight and limiting alcohol intake are particularly effective in controlling TGs concentrations. In addition, according to a scientific statement from the AHA, an increased physical activity may have positive effect on TGs, especially when combined with an adequate energy intake [40]. On the other hand, dietary influence on TGs levels is relatively small compared to lifestyle and genetic determinants. Current evidence suggests that dietary SFAs and cholesterol have little to no effect on TGs concentrations [41,42]. In turn, beneficial effects of plant protein, particularly from soy, on TGs levels have been observed in patients with chronic kidney disease not requiring dialysis [43]. As for fiber, while it is generally thought to improve the lipid profile, its effect on TGs is less consistent. An umbrella meta-analysis found no significant overall effect of fiber intake on TGs levels [44], but in certain populations, such as individuals with diabetes, or for specific fiber types, like β-glucans and galactomannans, a more pronounced TGs-lowering effect has been observed [44,45]. In our study, higher PC1 values were associated with elevated TGs and a dietary pattern resembling that observed in individuals with increased TC and LDL-C, with the result approaching statistical significance. However, given the existing literature, it is plausible that this finding reflects an overall, less favorable dietary and lifestyle pattern, rather than the direct impact of specific dietary components.

In terms of dietary intake, total energy consumption was comparable across dietary groups; however, OMNs consumed significantly more total protein than all other groups, while VEGs had the highest plant protein intake, significantly exceeding that of LOVs and OMNs. Moreover, VEG and LOV diets were characterized by significantly lower intakes of total fat, SFAs, and cholesterol compared with OMN diets, in line with previous reports [35,45,46]. These patterns were mirrored in the PCA results, which further highlighted OMN as the most distinct group, combining less favorable dietary features—such as the highest intake of animal protein, SFAs, and cholesterol, derived from animal products, particularly red meat [46], together with low fiber and plant protein consumption—with elevated biochemical markers, particularly TC and LDL-C. Additionally, PCA demonstrated a link between higher intake of energy from protein and fat—which is also characteristic of omnivorous diets [47,48]—and higher LDL-C levels. In contrast, greater dietary carbohydrate contribution was associated with lower concentrations of this lipoprotein, in line with earlier reports suggesting that diets higher in carbohydrates tend to have a more positive impact on LDL-C levels compared to higher intake of other macronutrients, highlighting that their dietary source is of key importance [49,50].

This consistent evidence underscores the adverse impact of omnivorous diets on lipid profile and presumably on cardiovascular risk. While the association between SFAs and unfavorable lipid profile is well-established [51,52,53], the impact of dietary cholesterol and animal protein remains inconsistent. Recent evidence suggests that while higher dietary cholesterol intake may lead to a slight increase in TC and LDL-C, this effect is relatively small and considerably less significant than that of SFAs and trans fatty acids (TFAs) [33,54]. In our study, OMN consumed more MUFAs than LOV and VEG—in line with some previous studies [48,55]—displaying less favorable lipid profile. While MUFAs are generally considered beneficial for cardiovascular health, it is crucial to consider their source—animal-derived products, such as high-fat dairy and processed meats, or plant foods, such as olives, nuts, seeds, or avocados and their oils [56]. In fact, replacing SFAs with plant-based MUFAs has been associated with lower total, cardiovascular, and certain cause-specific mortality, whereas MUFAs from animal sources were linked to higher mortality [57].

Red and processed meat consumption has been linked to elevated LDL-C, TC, and TGs concentrations, as well as an increased risk of CVDs, primarily due to the non-protein constituents, namely high SFA content [58]. Additionally, salt and nitrates are of concern: the former is positively associated with elevated blood pressure—a major risk factor for CVDs—while the latter promotes LDL-C oxidation, increasing damage of vascular endothelium and the risk of atherosclerosis [59,60]. Conversely, consuming lean meat, such as poultry and fish, appear to have more neutral, or in some cases even beneficial effects on cardiovascular health. Current recommendations suggest replacing red meat with poultry, fish, or plant-based protein sources (such as soy, other legumes, and nuts) to reduce cardiovascular risk [58,61,62]. Meta-analyses and randomized controlled trials consistently demonstrate that substituting plant protein for animal protein reduces LDL-C and TC levels [58,61,63,64]. However, the benefits are most pronounced when plant protein replaces red or processed meats, rather than fish or poultry. Additionally, isocaloric substitution of plant-based protein for animal protein lowers CVDs-related mortality [62], and substitution for processed meat protein is associated with lower CVDs risk factors, like BMI and blood pressure, and TGs levels [65]. In general, substitution of meat with minimally processed plant foods or appropriately formulated plant-based meat alternatives appears to confer benefits for lipid profile and broader cardiometabolic outcomes; nevertheless, confirmation through long-term studies remains necessary, especially for emerging plant-based meat products [66,67].

In several reviews and meta-analyses, higher dietary fiber intake, particularly water-soluble, viscous fiber—such as β-glucan, found in oats and barley—has been shown to reduce TC and LDL-C, with no significant effects on HDL-C and TGs [4,44]. These effects are thought to result from several mechanisms, including increased intestinal viscosity, bile acid binding, and modulation of the gut microbiota and lipid metabolism; namely, decreasing activity of HMG-CoA reductase, the key enzyme in endogenic cholesterol synthesis, and upregulating expression of the genes encoding LDL receptors, thus facilitating decomposition of this lipoprotein [68].

Importantly, a major source of water-soluble fiber and plant protein—both of which exert beneficial effects on lipid profile regulation—are legumes [69], which represent a crucial dietary component of plant-based eating patterns and are consumed in substantially greater amounts by vegetarians and vegans compared with omnivores [70,71].

In addition to ANOVA and PCA, the results of the multiple linear regression analysis also indicate that the omnivorous diet is the most distinct and exerts a significant effect on lipid levels, being associated with increases in TC, LDL-C, and TGs concentrations. Taken together, our findings reinforce the view that the omnivorous diet—typically higher in animal protein, SFAs, and dietary cholesterol—contrasts with plant-based diets, which are generally lower in these constituents and richer in fiber and plant protein, a combination that may contribute to the more favorable lipid profile observe in vegetarians and particularly vegans.

Alongside diet, physical activity is another important factor that may contribute to a more favorable lipid profile. In PCA, higher PC2 values—indicative of more physically active women—were associated with significantly lower TGs levels and higher HDL-C levels, consistent with previous findings. We also observed a clear trend toward higher TC and LDL-C concentrations in less physically active women, although this association did not reach statistical significance. However, a significant LDL-C–lowering effect of high physical activity was revealed in the regression model. Notably, sedentary behavior combined with obesity has been linked to increased TC and LDL-C levels, underscoring the role of exercise in CVDs prevention [72]. Conversely, in adults with metabolic syndrome, aerobic training has been shown to improve TC, LDL-C, HDL-C, and TGs levels, with evidence suggesting that greater training intensity and volume produce more pronounced benefits [73]. No significant association between physical activity and HDL-C was observed in our study. This finding may be attributed to the relatively young and metabolically healthy population. Additionally, the regression model explained only a small proportion of HDL-C variance, suggesting that other unaccounted-for factors—such as genetic influences—may have played a more prominent role. Nevertheless, it would appear that dietary interventions, such as plant-based eating patterns, combined with a regular physical activity, represent a synergistic approach to optimizing lipid profile and reducing cardiovascular risk.

Plant-based diets have gained an increasing popularity in recent years, particularly among women, motivated both by health considerations and by ethical concerns [74,75]. Indeed, women have largely driven the increase in vegetarianism over the past 15 years [71]. Given that CVD remain the leading cause of death in this population [2], it is crucial to investigate how plant-based eating patterns affect women’s cardiovascular health. Especially since an increasing number of publications highlight the problem of the striking underrepresentation of women in cardiovascular research [76,77], while it is known that the incidence and risk factor patterns of CVDs differ between the sexes [78]. Healthy plant-based diets based on unprocessed plant foods such as vegetables, fruits, legumes, whole grains, nuts, and seeds can be recommended as a strategy for CVDs prevention [79,80] as they are typically rich in plant protein, dietary fiber, MUFAs, PUFAs and phenolic compounds, and lower in SFAs and TFAs than a common Western diet [81]. Moreover, these diets are associated with improvements in key risk factors for CVDs—reduced blood pressure, lower BMI, more favorable lipid profile, and decreased systemic inflammation [14]. However, when such diets are dominated by ultra-processed foods, rich in refined carbohydrates, SFAs, TFAs and other harmful components, they still may lead to unfavorable lipid profiles despite animal product avoidance [82,83].

Sisay et al. [84] showed that even short-term adherence to a vegan diet (7 weeks) can lead to significant reductions in blood pressure, body mass, BMI, TC, LDL-C, HDL-C, and TC:HDL-C ratios. Notably, these beneficial effects were more pronounced in women compared to age-matched men [84]. This may be particularly important for older women, as CVDs risk increases with age. It appears plausible that plant-based dietary patterns may confer similar beneficial effects on lipid profile and overall cardiovascular health in postmenopausal women as those observed in younger populations [29,85,86,87]. Menopause marks a significant transition in women’s health, bringing not only hormonal changes but also a range of metabolic alterations, including increased body mass, accumulation of visceral fat, reduced insulin sensitivity, elevated systolic blood pressure, and changes in circulating lipid levels [88]. Nevertheless, evidence specific to this population remains limited, and further targeted, long-term studies are warranted to clarify the role of healthy plant-based diets in both prevention and management of CVDs in women after menopause [89,90].

A major strength of our study was the focus on a group of women—underrepresented in cardiovascular health research—that was well-defined and homogeneous, reducing the influence of potential confounders such as age and BMI. In addition, the prospective assessment of dietary intake using 7-day food diaries, verified with the dietitian during in-person interview, minimized recall bias and ensured greater accuracy compared with often used food frequency questionnaires or 24 h dietary recalls. This approach also allowed for a more detailed evaluation of dietary composition, including specific nutrient intakes rather than only general dietary patterns. Therefore, our study not only examined the impact of diet itself but attempted to connect intake of specific food components with the circulating lipid levels. Nevertheless, it has some limitations. First, the size of two analyzed diet groups was relatively small. Second, online recruitment may have attracted more health-conscious individuals. Third, self-reported data, like physical activity level, may be subject to a recall bias. Fourth, we studied only young women. Fifth, information on contraceptive use, which may have had a minor influence on lipid levels, was not collected. Therefore, there is a strong need for further research encompassing other populations, particularly peri- and postmenopausal women and individuals with dyslipidemia.

## 5. Conclusions

The advantage of our work lies in presenting results from a homogeneous group of participants with respect to sex, age, BMI, and body fat content, and in comparing the lipid profile not only between dietary groups but also in relation to actual intake of energy and different nutrient types. The results indicate a potential cardioprotective effect of vegetarian and vegan diets, especially in the context of primary prevention in young, healthy women, suggesting that lower intake of SFAs, cholesterol, and animal protein, together with higher intake of fiber and plant protein, exerts the strongest influence on lipid profile, particularly TC and LDL-C levels. The findings may be of practical relevance for developing dietary recommendations and public health strategies for young women. Nevertheless, future studies involving larger cohorts—particularly well-designed intervention trials—are warranted to further elucidate the impact of plant-based diets on cardiovascular risk, especially in specific populations such as peri- and postmenopausal women and individuals with dyslipidemia.

## Figures and Tables

**Table 1:** Comparison of basic characteristics in different diet groups. Data are shown as mean ± standard deviation and were analyzed using one-way ANOVA (BMI, body fat) or median ± quartile deviation and analyzed using Kruskal–Wallis test and Dunn’s test (age, time on diet, WHR). * Groups with statistically significant difference. VEG—vegan, LOV—lacto-ovo vegetarian, PSC—pescatarian, OMN—omnivore, BMI—body mass index, WHR—waist-to-hip ratio.

Variable	Diet Type				*p*
	VEG (*n* = 45)	LOV (*n* = 52)	PSC (*n* = 12)	OMN (*n* = 19)	
Age (y)	24.0 ± 4.5	26.0 ± 3.5	26.5 ± 6.7	31.0 ± 6.0	0.14
Time on diet (y)	2.2 ± 1.1	2.8 ± 2.8	3.4 ± 2.9	n/a	1.00
BMI (kg/m^2^)	21.0 ± 1.6	21.40 ± 1.79	22.05 ± 2.02	21.55 ± 1.74	0.28
Body fat (%)	24.4 ± 3.85	24.95 ± 3.61	24.77 ± 3.47	24.77 ± 3.02	0.93
WHR	0.763 ± 0.025	0.749 ± 0.031 *	0.767 ± 0.033	0.796 ± 0.026 *	0.02

**Table 2:** Serum levels of lipid profile components. VEG—vegan, LOV—lacto-ovo vegetarian, PSC—pescatarian, OMN—omnivore. Means were compared using one-way ANOVA (HDL-C) and Welch’s ANOVA (LDL-C, TC) followed by the Games–Howell test. * Data are shown as median ± quartile deviation and were analyzed using the Kruskal–Wallis test and Dunn’s test.

Lipid Profile	Diet Type	Mean ± SD	95% CI	*n*	*p*					
					VEG vs. LOV	VEG vs. PSC	VEG vs. OMN	LOV vs. PSC	LOV vs. OMN	PSC vs. OMN
HDL-C	VEG	74.27 ± 20.65	67.99–80.55	44	0.447					
	LOV	80.09 ± 21.88	73.87–86.31	51						
	PSC	82.02 ± 15.49	72.18–91.86	12						
	OMN	81.63 ± 25.68	69.25–94.01	19						
LDL-C	VEG	69.04 ± 24.67	61.45–76.63	43	0.754	0.808	<0.001	0.983	<0.001	0.028
	LOV	76.65 ± 28.60	68.60–84.69	51						
	PSC	82.02 ± 16.57	71.49–92.56	12						
	OMN	122.43 ± 68.10	89.60–155.25	19						
TC	VEG	162.34 ± 20.81	156.02–168.67	44	0.490	1.0	0.007	0.914	0.093	0.052
	LOV	169.03 ± 19.85	163.45–174.62	52						
	PSC	163.09 ± 17.63	151.89–174.29	12						
	OMN	186.20 ± 32.39	170.09–202.30	18						
TGs *	VEG	105.78 ± 24.89	91.78–116.67	43	1.0	1.0	0.046	1.0	0.083	0.042
	LOV	108.89 ± 19.44	99.56–121.33	49						
	PSC	93.33 ± 22.17	82.44–126.78	12						
	OMN	137.48 ± 12.44	122.89–147.78	16						

**Table 3:** Daily intake of energy and selected nutrients. VEG—vegan, LOV—lacto-ovo vegetarian, PSC—pescatarian, OMN—omnivore. Means were compared using one-way ANOVA (fats, MUFAs, plant protein, fiber) followed by Tukey’s test and Welch’s ANOVA (total energy, protein, animal protein, SFAs, PUFAs, cholesterol, carbohydrates) followed by the Games–Howell test.

Nutrient Intake	Diet Type	Mean ± SD	95% CI	*n*	*p*					
					VEG vs. LOV	VEG vs. PSC	VEG vs. OMN	LOV vs. PSC	LOV vs. OMN	PSC vs. OMN
Total energy (kcal)	VEG	2001 ± 458	1861.99–2140.53	44	0.112					
	LOV	1824 ± 309	1731.09–1917.04	45						
	PSC	1935 ± 261	1760.45–2110.90	11						
	OMN	1999 ± 338	1835.59–2161.93	19						
Protein (% of energy)	VEG	12.2 ± 1.8	11.66–12.81	42	0.393	0.196	<0.001	0.659	<0.001	<0.001
	LOV	13.4 ± 1.9	12.79–13.96	45						
	PSC	14.9 ± 2.8	13.17–16.67	12						
	OMN	20.5 ± 6.9	17.14–23.77	19						
Animal protein (g)	VEG	0.0 ± 0.0	not applied	45	<0.001	<0.001	<0.001	0.220	<0.001	<0.001
	LOV	16.2 ± 10.3	13.10–19.23	46						
	PSC	24.6 ± 12.4	16.68–32.46	12						
	OMN	51.4 ± 19.6	41.91–60.80	19						
Plant protein (g)	VEG	44.0 ± 14.0	39.70–48.34	43	<0.001	0.110	0.007	0.761	0.895	0.984
	LOV	30.8 ± 11.0	27.56–34.01	47						
	PSC	34.7 ± 11.7	26.87–42.62	11						
	OMN	33.1 ± 9.7	28.44–37.75	19						
Fats (% of energy)	VEG	29.1 ± 6.0	27.24–30.87	44	0.030	0.126	<0.001	0.974	0.027	0.321
	LOV	32.0 ± 4.4	30.75–33.32	48						
	PSC	32.7 ± 5.4	29.32–36.16	12						
	OMN	36.0 ± 4.3	33.89–38.04	19						
SFAs (g)	VEG	13.4 ± 5.6	11.72–15.16	43	0.006	0.062	<0.001	0.839	0.064	0.705
	LOV	18.0 ± 5.7	16.34–19.70	47						
	PSC	20.3 ± 6.2	16.12–24.44	11						
	OMN	23.2 ± 9.2	18.79–27.62	19						
MUFAs (g)	VEG	19.2 ± 6.9	17.06–21.37	42	0.670	0.277	<0.001	0.680	0.004	0.454
	LOV	20.8 ± 6.7	18.89–22.74	49						
	PSC	23.3 ± 6.5	18.94–27.69	11						
	OMN	27.1 ± 6.4	24.00–30.15	19						
PUFAs (g)	VEG	16.5 ± 7.2	14.38–18.70	45	<0.001	0.323	0.156	0.964	0.868	1.0
	LOV	11.3 ± 4.5	9.98–12.63	47						
	PSC	12.4 ± 4.4	9.46–15.44	11						
	OMN	12.7 ± 4.0	10.77–14.63	19						
Cholesterol (mg)	VEG	0.0 ± 0.0	not applied	45	<0.001	<0.001	<0.001	0.476	<0.001	0.377
	LOV	160.5 ± 111.8	128.03–192.94	48						
	PSC	220.5 ± 105.1	149.94–291.13	11						
	OMN	287.6 ± 158.5	208.74–366.36	18						
Carbohydrates (% of energy)	VEG	56.6 ± 5.5	54.86–58.30	42	0.026	0.175	0.185	0.951	0.999	0.978
	LOV	52.3 ± 5.7	50.69–53.96	49						
	PSC	50.8 ± 5.2	47.52–54.15	12						
	OMN	52.0 ± 11.8	46.07–57.85	18						
Fiber (g)	VEG	44.4 ± 15.1	39.76–48.96	44	<0.001	0.207	0.018	0.754	0.951	0.964
	LOV	31.3 ± 12.5	27.74–34.92	49						
	PSC	35.7 ± 10.8	28.79–42.55	12						
	OMN	33.3 ± 13.8	26.61–39.95	19						

**Table 4:** Results of OLS regression analyses examining associations between lipid parameters and the following predictors: diet type (omnivorous vs. non-omnivorous), BMI (kg/m^2^), age (y), total energy intake (kcal), physical activity level (high vs. low). F—F-test for overall model significance; β—unstandardized regression coefficient, representing the expected change in the dependent variable per unit change in the predictor; SE—standard error of the coefficient; t—Student’s *t*-test for each coefficient; R^2^—proportion of variance in the dependent variable explained by the model; adjusted R^2^—R^2^ adjusted for the number of predictors.

Lipid Profile	F	β ± SE	t	R^2^	Adjusted R^2^
HDL-C	1.40; *p* = 0.23	No significant predictors		0.061	0.017
LDL-C	8.14; *p* < 0.001	Diet (omnivorous): 45.85 ± 9.05	5.07; *p* < 0.001	0.274	0.240
		Total energy intake: −0.019 ± 0.009	−2.10; *p* = 0.038		
TC	7.451; *p* < 0.001	BMI: 2.42 ± 1.18	2.06; *p* = 0.042	0.255	0.221
		Age: 1.09 ± 0.26	4.00; *p* < 0.001		
		Physical activity (high): −12.65 ± 6.25	−2.03; *p* = 0.045		
		Diet (omnivorous): 16.37 ± 5.52	2.97; *p* = 0.004		
TGs	2.635; *p* = 0.028	Diet (omnivorous): 19.35 ± 8.32	2.35; *p* = 0.021	0.112	0.070

## Data Availability

The data that support the findings of this study are available from the corresponding author upon a reasonable request. However, the dataset is part of an ongoing research project and is not publicly available at this time.
